# Network Analysis Identifies Phase Transitions for Tumor With Interacting Cells

**DOI:** 10.3389/fphys.2022.865561

**Published:** 2022-07-01

**Authors:** Amir Hossein Darooneh, Mohammad Kohandel

**Affiliations:** ^1^ Department of Applied Mathematics, University of Waterloo, Waterloo, ON, Canada; ^2^ Department of Physics, University of Zanjan, Zanjan, Iran

**Keywords:** tumor growth, metastasis, Brownian dynamics (BD), interacting cells, network analysis, phase transition

## Abstract

Metastasis is the process by which cancer cells acquire the capability to leave the primary tumor and travel to distant sites. Recent experiments have suggested that the epithelial–mesenchymal transition can regulate invasion and metastasis. Another possible scenario is the collective motion of cells. Recent studies have also proposed a jamming–unjamming transition for epithelial cells based on physical forces. Here, we assume that there exists a short-range chemical attraction between cancer cells and employ the Brownian dynamics to simulate tumor growth. Applying the network analysis, we suggest three possible phases for a given tumor and study the transition between these phases by adjusting the attraction strength.

## 1 Introduction

Metastasis is a critical and complex phenomenon in the biology of cancerous tumors. Despite many findings of genetics and epigenetics of cancer cells, biochemistry of intracellular organelles, biomechanical properties of cytoskeleton, and tumor microenvironment, there are still a collection of unanswered questions on how cancer spreads in the body. Two main scenarios have been proposed for metastasis, the epithelial–mesenchymal transition (EMT) and collective cell motions. An epithelial cell undergoes changes in morphology and phenotype through EMT. It loses its polarity and cell–cell adhesion and breaks through the connections with the underlying membrane. These changes cause the cells to become mesenchymal with high migration characteristics and invasive properties (Hanahan and Weinberg, 2000; Chaffer and Weinberg, 2011; Ribatti et al., 2020). The EMT scenario is supported by many experimental and computational works. Here, we only refer to a limited number of them (Nouri et al., 2014; Yeung and Yang, 2016; Pal et al., 2019; Xing and Tian, 2019). Another approach is based on the collective migration of clusters of cells which was observed experimentally and studied by simulation for some types of cancer cells (Friedl et al., 1995; Angelini et al., 2010; Angelini et al., 2011; Park et al., 2015; Cheung and Ewald, 2016; Mitchel et al., 2020). Sadati et al. (2013) have provided explanations on what is happening in terms of physical forces, and for the first time, they proposed the idea of jamming–unjamming transition for metastasis. Later, experiments revealed that the epithelial cells form clusters in culture and become coarse by increasing the density. The epithelial cells show unjammed–jammed transition, in contrast to the mesenchymal cells, which disperse individually in the environment (Castro et al., 2016). The evidence which comes from the analysis of patient samples, as well as experimental models, shows that the tumor cells migrate in clusters (Cheung and Ewald, 2016). It is now accepted that a connection exists between the unjamming transition of cancer cells and tumor progression, at least for some kinds of cancer cells (Oswald et al., 2017). A recent study by a group of scientists shows the tumor invasion may be switched from the cellular cluster to the individual cell scenario, depending on the substrate characteristics and cell type (Kang et al., 2020). Lately, a physical model has been proposed by Glimson and Golestanian that relies on the assumption that a chemical long range interaction exists between the cancer cells (Gelimson and Golestanian, 2015). The chemical attraction between the cells leads to coagulation, while the diffusion mechanism behaves in the opposite way. The diffusion wins the competition for weak chemical fields, and the system of cells experiences a second-order phase transition to the metastatic state.

Here, we assume a short-range chemical attraction between the cancer cells and employ the Brownian dynamics to simulate tumor growth. The network theory is used to analyze the results. Three phases of a tumor are distinguished, and the transition between these phases is observed by adjusting the attraction strength.

This article is organized as follows. In the next section, we present the mathematical framework for our simulation, including a description of the Brownian dynamics and modelling the chemical interaction between the cells in two dimensions. The simulation procedure is described in detail in the third section. The fourth section is devoted to introducing some basic concepts from the network theory that we use in analyzing the results. We present our simulation results in the fifth section. These results are discussed in the final section.

## 2 Methods

### 2.1 Motion of Cancer Cells in the Extracellular Matrix

The extracellular matrix (ECM) provides structural support as well as the physical environment for cell activity. It also facilitates the transfer of essential chemicals to cells. The ECM is composed of protein fibers that are embedded in water, in addition to polysaccharides. The composition of ECM is not definite, and it varies in different tissues. This causes ECM to have various physical and topological properties. ECM has a dynamic heterogeneous structure that affects its mechanical properties. Cells attach to this structure by using some receptors, e.g., integrins. These receptors are involved in cell migration through the ECM (Uzman et al., 2003; Bonnans et al., 2014).

It is an experimentally accepted fact that the cancer cells, like the other cells, move randomly on the ECM (Selmeczi et al., 2008; Flate and Stalvey, 2014; Wu et al., 2014; Huda et al., 2018; Safaeifard et al., 2018; Kwon et al., 2019). The randomness in cell motion could be due to the random direction of ECM protein fibres or even intracellular substructure changes, leading to different types of locomotion of cells (Huber et al., 2013). There are several models proposed for the description of cancer cells’ random motion (Huda et al., 2018; Kwon et al., 2019). Since different cell lines’ behavior is not the same, even each cell line may have distinct behavior in short and long time scales (Huda et al., 2018; Kwon et al., 2019). Most of the models which describe the free motion of cells are reduced to a simple random walk in a long time period. In addition to random motion, a cell can have directional migration. The presence of external stimuli brings about a bias in the motion of cells toward the source of stimuli (Eisenbach, 2004; Gautreau, 2018).

Tumor growth is a long-time process in which the cancer cells move in a viscous medium of ECM and may be affected by chemical signals. Therefore, it is rational to use the Brownian dynamics for the evolution equation of cancer cells (Drasdo and Höhme, 2005; Klank et al., 2018; Bull et al., 2020).

$$\frac{dr_{i}\left(t\right)}{dt}=\frac{1}{\nu }F\left(r_{i}\right)+\sqrt{2D}\;\eta_{i}\left(t\right).$$ (1)

Here, **r**_*i*_(*t*) is the position of the *i*-th cell at time *t*, the external force exerted on this cell is shown by **F**(**r**_*i*_), *ν* is the friction coefficient and depends on the cells’ geometry and viscosity of ECM, *D* is the diffusion coefficient of cell, and ***η***_*i*_(*t*) is a Gaussian random vector.

$$\begin{array}{cc} <\eta \left(t\right)> & =0\\ <\eta_{i}\left(t\right)\cdot \eta_{j}\left(t^{\prime }\right)> & =\delta_{i,j}\delta \left(t-t^{\prime }\right) \end{array}.$$ (2)

In this study, we assume that all the cancer cells secrete chemicals to attract each other. This type of interaction between the same cells is observed for bacteria (Eisenbach, 2004) and neural stem cells (Ladewig et al., 2013). It is also used for the justification of the collective migration of cells (Camley et al., 2016). The motion of any cell is impacted from the gradient of chemicals that other cells have secreted.

$$F\left(r_{i}\right)=-\kappa \underset{j\neq i}{\sum }\nabla_{i}\varphi \left(\left|r_{i}-r_{j}\right|\right),$$ (3)

where *φ*(**r**, *t*) is the concentration of chemicals or as we call it, the chemical potential at point **r** in time *t* and *κ* represents the strength of force.

We can obtain the chemical potential from the diffusion equation.

$$\frac{1}{D_{c}}\frac{\partial \varphi \left(r,t\right)}{\partial t}=\nabla^{2}\varphi \left(r,t\right)+\alpha_{0}\underset{j}{\sum }\delta \left(r-r_{j}\left(t\right)\right)-\lambda \varphi \left(r,t\right).$$ (4)

Here, *D*_*c*_ stands for the diffusion coefficient of chemicals. The diffusion coefficient, *D*_*c*_, for macromolecules and proteins is about 10^2^ (*μm* ^2^/*s*) (Milo et al., 2009). Every cell can be considered as the point source of chemicals, *α*_0_ is the rate of the emitting chemical by a cell divided by the diffusion coefficient of chemicals. In the same manner, the rate of disappearance of chemicals in the environment divided by the chemical diffusion coefficient is *λ*.

The chemical diffuses rapidly through the ECM in comparison to the cell’s movement. It means that any chemical disturbance in the ECM rapidly spreads, and the chemical potential becomes stationary before the cells have considerable displacement. Hence, it is rational to use the stationary diffusion equation to obtain the chemical potential for any configuration of cells.

$$-\nabla^{2}\varphi \left(r\right)=\alpha_{0}\underset{j}{\sum }\delta \left(r-r_{j}\right)-\lambda \varphi \left(r\right).$$ (5)

The abovementioned equation is similar to Poisson equation for an external point charge in the plasma. Hereafter, we proceed with the result in two dimensions (Liebchen and Löwen, 2019).

$$\varphi \left(r\right)=\alpha_{0}\underset{j}{\sum }K_{0}\left(\sqrt{\lambda }|r-r_{j}|\right),$$ (6)

where *K*_0_(*r*) is the modified Bessel function of the second kind order zero. The chemical force on the *i*-th cell can be calculated by using Eq. 3 for the abovementioned potential.

$$F\left(r_{i}\right)=\kappa \alpha_{0}\sqrt{\lambda }\underset{j\neq i}{\sum }K_{1}\left(\sqrt{\lambda }|r_{i}-r_{j}|\right)\frac{r_{i}-r_{j}}{\left|r_{i}-r_{j}\right|},$$ (7)

where *K*_1_(*r*) represents the modified Bessel function of the second kind order one.

The evolution equation for the system of interacting cells on the ECM is obtained by putting the abovementioned result in Eq. 1.

$$\frac{dr_{i}\left(t\right)}{dt}=\mu \alpha_{0}\sqrt{\lambda }\underset{j\neq i}{\sum }K_{1}\left(\sqrt{\lambda }|r_{i}-r_{j}|\right)\frac{r_{i}-r_{j}}{\left|r_{i}-r_{j}\right|}+\sqrt{2D}\;\eta_{i}\left(t\right),$$ (8)

where *μ* = *κ*/*ν* is called the cell motility, and it shows the capacity of cells for motion on the ECM.

Tumor growth is a long-run process compared with the cell proliferation cycle. Therefore, we should take into account the birth and death events for cells during the growth process. The following subprocesses must be considered with Equation 8 to fully describe the growth process:

$$\left\{\begin{array}{c} C\overset{\Gamma }{\to }C+C\;\\ C\overset{\gamma }{\to }\emptyset \; \end{array}.\right.$$ (9)

Here, Γ and *γ* are the birth and death rates, respectively. To have an increasing number of cells in the tumor over time, Γ must be greater than *γ*.

### 2.2 Simulation Procedure

The simulation is an affordable way for finding the solution of stochastic differential equations. In this regard, we divided the processing time into the number of time steps. We use the linear approximation to find the solution at any step, which plays the role of the initial condition for the equation in the next step. Here, we use the Euler–Maruyama method (Kloeden and Platen, 1992) to discretize Eq. 8.

$$r_{i}\left(t+\delta t\right)=r_{i}\left(t\right)+\mu \alpha_{0}\sqrt{\lambda }\overset{N}{\underset{\begin{array}{c} j=1\\ j\neq i \end{array}}{\sum }}K_{1}\left(\sqrt{\lambda }|r_{i}-r_{j}|\right)\frac{r_{i}-r_{j}}{\left|r_{i}-r_{j}\right|}\delta t+\sqrt{2D\;\delta t}\;\eta_{i}\left(t\right),$$ (10)

where *δt* is the size of the time step.

A suitable system of units should be used to avoid dealing with the large or small numbers in simulations. Here, we choose the cell’s proliferation cycle, *τ* = 10^4^ *s*, and diameter of the cell, *ℓ* = 10 *μm*, as the unit of time and length, respectively. It is better to write Eq. 8 in its dimensionless form. By defining the attraction range, $\sigma =1/\left(\ell \sqrt{\lambda }\right)$ and the attraction strength Δ = *μα*_0_ *τ*/*ℓ* ^2^, we arrive at the following equation:

$$r_{i}\left(t+\delta t\right)=r_{i}\left(t\right)+\frac{\Delta }{\sigma }\overset{N}{\underset{\begin{array}{c} j=1\\ j\neq i \end{array}}{\sum }}K_{1}\left(\frac{\left|r_{i}-r_{j}\right|}{\sigma }\right)\frac{r_{i}-r_{j}}{\left|r_{i}-r_{j}\right|}\delta t+\sqrt{2\;\delta t}\;\eta_{i}\left(t\right).$$ (11)

In the abovementioned equation, we assume *D* = 10^−2^ *μm* ^2^/*s*, it is a good approximation for the diffusion coefficient of cancer cells (Milo et al., 2009; Franssen et al., 2019).

It is worth noting that in terms of the new time unit, the birth rate Γ is equal to one, and for the cancer cell death rate, we use the value *γ* = 0.1. The tumor growth rate can be controlled by adjusting the value of *γ*, but here we intended to study the tumour growth qualitatively.

The simulation starts by locating a cell at the origin, the cell will move when a birth event happens. In each time step of the simulation, we first examine whether each cell dies or not. This is easily done by comparing a uniform random number between zero and one with the product of the death rate and the time step’s size. If a cell dies, we remove it from the system. In the later stage, we allow the remaining cells to randomly proliferate in the same way as the previous stage. The newborn cell is randomly located adjacent to its mother, it is sufficient to select a uniform random number *θ* between zero and 2*π* . Then, the coordinates of the daughter cell are *x*_*daughter*_ = *x*_*mother*_ + cos(*θ*) and *y*_*daughter*_ = *y*_*mother*_ + sin(*θ*). In the third stage, all the cells in the system change their position according to Eq. 11, one by one. In this stage, we must compute the chemical force acting on each cell and draw a random vector from a Gaussian distribution as a random force exerted on the cell. The simulation may end at early stages due to the death of all cells. For the statistical results, we take into account only simulations that have a nonzero number of cells at the end.

In our simulation, the cells are not point particles and have finite sizes. To be specific, in two dimensions, they are disced with a diameter equal to one. In the birth event or changing the cell’s position, some overlapping may occur. In that case, we should repeat the procedure again to find a new position without any overlapping. If this situation does not occur for a cell after a number of times, e.g., 100, we put its position unchanged in the system, or we do not accept the birth of a new cell. It is worth noting that at each step we update the cells one after the other. Despite the fact, overlapping less than 0.2 of cell size is acceptable because, in reality, the cells are compressible objects (Drasdo and Höhme, 2005).

In our simulation, we deal with several parameters, but most of them are fixed, namely, *D* = 1, Γ = 1, *γ* = 0.1, *δt* = 0.001 and number of steps is 40,000. In this study, we fix the attraction range, *σ* = 1.5. Then, the attraction strength, Δ is the only free parameter of the problem. For each value of Δ, we run the simulation 20 times to be ensured that the result is correct and obtain the statistical error.

### 2.3 Network Analysis

The idea of utilizing the network theory for describing the complex phenomena has been used for many years (da Fontoura Costa et al., 2011; Estrada, 2011; Barabási, 2016; Latora, 2017). Recently, it has also appeared as a powerful tool for studying the granular material and exploring its underlying physics (Papadopoulos et al., 2018). Granular material is a system of grains or particles with finite size. They can interact with each other only if they are in contact. Friction between the grains and thermal fluctuations determines their organization in the system. This is similar to the situation that we have in the system of cancer cells or tumors, and it allows us to use the network theory with a minor modification in analyzing the tumor structure.

**FIGURE 1:**
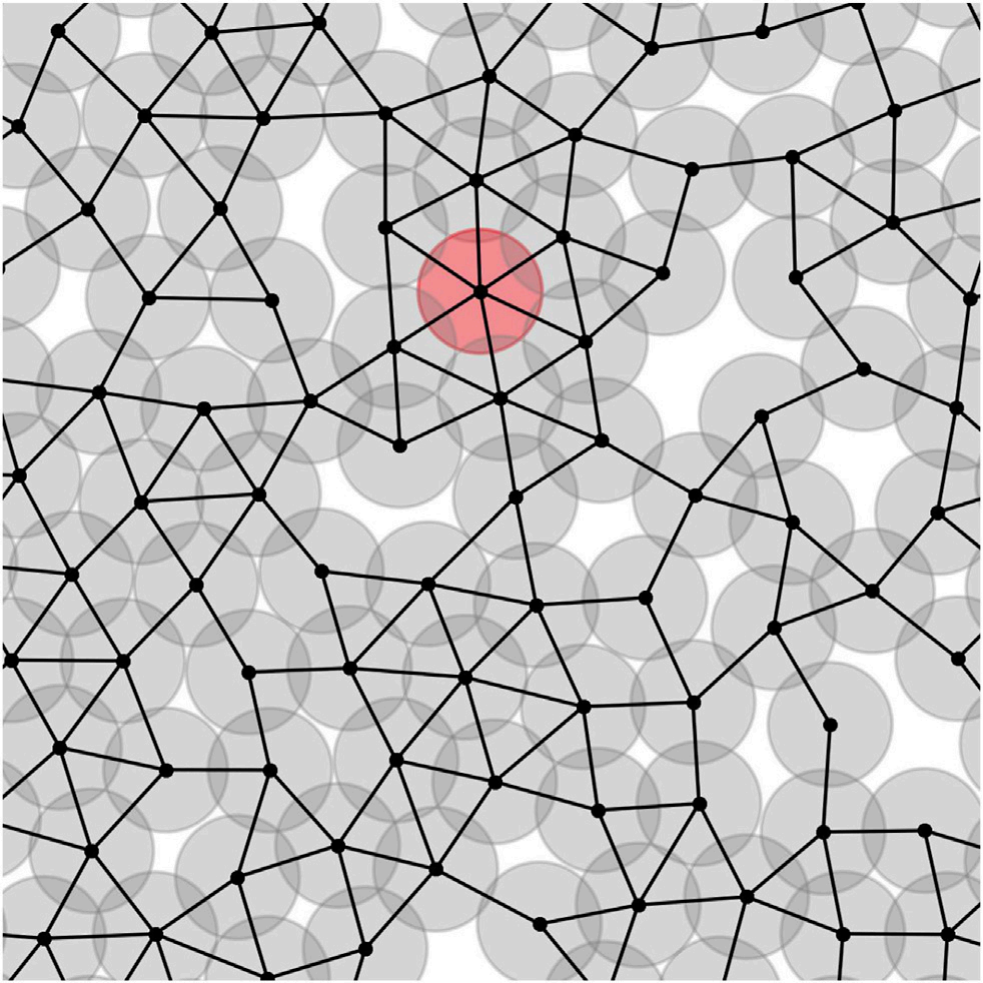
Part of the network associated with a tumor. The center of cells are the network nodes. If two cells are in contact, a link is formed between their centers. The red circle shows an instance cell with maximum number of neighbors, *k* *_max_* = 6.

We can assign a network to each tumor. The center of cells plays the role of nodes, and they are linked to each other if respected cells are in contact. It means that their centers’ distances are less than or equal to one. Figure 1 depicts the part of a tumour network. The circles are the cells, and dark parts mean overlapping between the cells which demonstrates compression of cells. The red circle is an instance node with a maximum number of neighbors. For the sake of having a real sight, we depict a tumor and its associated network in a unique picture, Supplementary Figure S1.

The degree of a node is defined as the number of links it has to other nodes. In a two-dimensional contact network, the degree varies between *k* *_min_* = 1 and *k* *_max_* = 6. It should be mentioned that the isolated nodes are not considered as a part of the network. There is a quantity that could be useful in analyzing the networks, *P*_*k*_ displays the number of nodes with degree *k* to the total number of nodes. It can also be interpreted as the probability of finding a node with degree *k* in the network. The behavior of *P*_*k*_ in terms of *k* is an important measure as well. It encompasses information about the structure of the network, and many other quantities are related to it. As an instance, the average degree is defined as $<k>=\sum_{k}kP_{k}$ , while it is calculated directly by

$$<k>=\frac{1}{N}\overset{N}{\underset{i=1}{\sum }}k_{i},$$ (12)

where *N* is the number of nodes and *k*_*i*_ is the degree of the *i*-th node. It is worth noting that the sum of nodes’ degrees is twice the number of links, *L*.

To show the dependence of degree of nodes to their neighbor degrees, we can define a measure which is called the average nearest neighbors degree, *K*_*nn*_.

$$K_{nn}=\frac{1}{N}\underset{i}{\sum }\frac{1}{k_{I}}\underset{j\in N_{i}}{\sum }k_{j},$$ (13)

where $N_{i}$ represent the set of neighbors of the *i*-th node. This measure gives us another piece of information about the structure of the network.

Transitivity or global clustering coefficient estimates the closeness of nodes in a contact network as a whole. It is defined as

$$T=\frac{N_{△}}{N_{\wedge }},$$ (14)

where *N*_△_ is the number of existent triangles and *N*_∧_ represents the number of possible triangles. Three neighboring nodes with two links is a possible triangle while the existing triangle has three links.

The clustering coefficient of a node demonstrates how many of its neighbors are connected to each other

$$C_{i}=\frac{2L_{i}}{k_{i}\left(k_{i}-1\right)},$$ (15)

where *k*_*i*_ is the degree of the *i*-th node and *L*_*i*_ stands for the number of links between its neighbors. It is evident that 0 ≤ *C*_*i*_ ≤ 1. The average of this quantity over the network identifies the local clustering feature of the network.

$$<C>=\frac{1}{N}\overset{N}{\underset{i=1}{\sum }}C_{i}.$$ (16)

It is a parameter for showing the clustering behavior of the network beside the transitivity.

A network is connected if every node is reachable from another node by traversing the links. In the otherwise network, it is called disconnected and consists of more than one component. The number of components *N*_*c*_, their size distribution *p*(*s*), and the largest size component *S* *_max_* are important features of a network. The size of a component is the number of nodes that it has. For computing these quantities, we use the breadth-first search method (Kozen, 1992; Erickson, 2019).

To find the other properties of networks, we refer the readers to many textbooks which cover these topics.

## 3 Results

Solid, liquid, gas, and phase transition, all of these concepts are defined in the equilibrium thermodynamics where we have an infinite system that its properties at the macroscopic level do not change with time. In tumor growth, we deal with a finite system that continuously is in nonequilibrium condition. However, it is possible to assign a phase to a tumor by considering only its spreading feature. A benign tumor is the accumulation of cells without the ability to spread, like a solid. Although several reasons exist to describe the malignancy of a tumor, we focus on its mechanical reason. When a tumor is malignant, the adhesion between the cells is decreased (Kim et al., 2019), then some groups of cells may be separated from the tumor and invade the adjacent tissues in the presence of external chemical stimulation. Therefore, we can assume that a tumor with many components can be considered malignant.

The chemical force between the cells is short-range falls exponentially as the distance between them increases, so for each configuration of cells, we can approximate the energy of the system by the number of pairs in contact and neglecting the contribution of the other pairs. This is why we choose the number of links or equivalently the average degree as the thermodynamic energy. The parameter Δ appears as a coefficient in the chemical force and then in the energy. Thus, its inverse can be considered as temperature. The changes in the specific heat, i.e. the derivative of energy with respect to the inverse of Δ determines the nature of transition as well as the transition point. Abrupt change of specific heat in the vicinity of a point is the characteristic of a first-order phase transition, while the existence of a peak in specific heat shows a phase transition of a second-order type. Indeed, in the second-order phase transition, the specific heat must be infinite, but it turns out to be a peak for the finite systems. Although we talk about gas, liquid, and solid states for a tumor, these states change among each other by a second-order phase transition.

**FIGURE 2:**
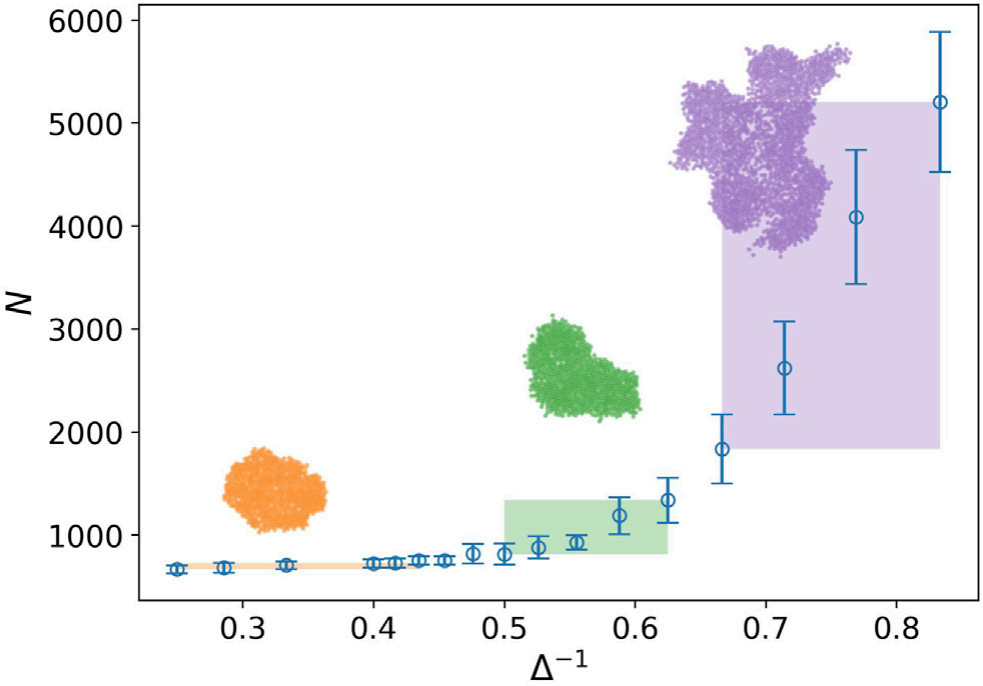
Number of nodes against the inverse of attraction strength (blue circle), and the attraction range has a fixed value, *σ* = 1.5. The error bars are the standard deviation of sampling over 20 simulation runs. Three regions with distinct behaviors are identified by the rectangles. The rectangle length approximately determines the domain of the phase and its height shows variation of the tumor size in that domain. The snapshot of a sample tumor is also illustrated for each region.

In Figure 2, the behavior of the number of nodes is plotted against the inverse of attraction strength i.e., Δ^−1^. The attraction range has a fixed value, *σ* = 1.5. The number of nodes or the network size represents the tumor size as well. Three regions are clearly observed in this plot. In the first region, we do not have any considerable change in the size of the tumor. It is the closed packed state or a solid tumor. In the next region, even though the tumor size grows by increasing the inverse of attraction strength, this is not a substantial rise. Eventually, we observe a drastic change in the tumor size in the third region. The snapshot of a sample tumor is also illustrated for each region. The same behavior can be seen in the plot of the number of components. These regions can be interpreted as different phases for a tumor. To be more accurate, we should find some quantities, which demonstrate the phases change and also allow us to estimate the transition point.

**FIGURE 3:**
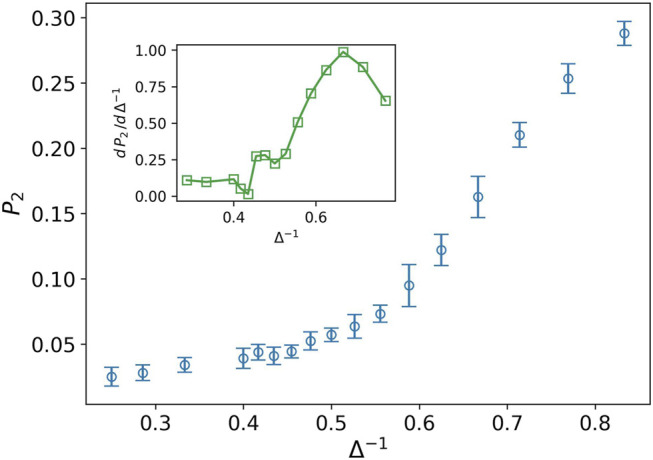
Fraction of nodes with degree two *P*_2_ (blue circle) against the inverse of attraction strength, and the attraction range has a fixed value, *σ* = 1.5. The error bars are the standard deviation of sampling over 20 simulation runs. The increasing behavior means more average distance between the cells in tumor. The inset plot displays a derivative of *P*_2_ as well. The peak splits liquid-like and gas-like phases.

While the distance of the cells is more than one, most of the cells cannot connect to each other in the language of network theory. Hence, we expect to find more small clusters in the tumor network. The nodes in these clusters have a small degree as well. By increasing the attraction strength, the average distance between the cells decreases and the small clusters stick together and form the larger size clusters. Consequently, the number of nodes with a high degree increases in contrary to the small degree nodes. The quantity *P*_2_, illustrates this aggregation phenomenon. Figure 3 shows the increasing behavior of *P*_2_ against the inverse of attraction strength. The inset plot also displays the derivative of *P*_2_, which has a peak at Δ^−1^ ∼ 0.66. The existence of peak means that we have two phases on different sides of the peak. On the left side, *P*_2_ rapidly grows while the speed of growth decreases quickly after the peak. The shape of this curve reminds us of the behavior of heat capacity against temperature during phase transition in magnetic nanoparticles (Kapusta et al., 2018). In the above-explained scenario, the cells or small clusters are apart from one another. By increasing the attraction strength, they come to get closer to each other. This is like the condensation from a gaseous to a liquid phase.

**FIGURE 4:**
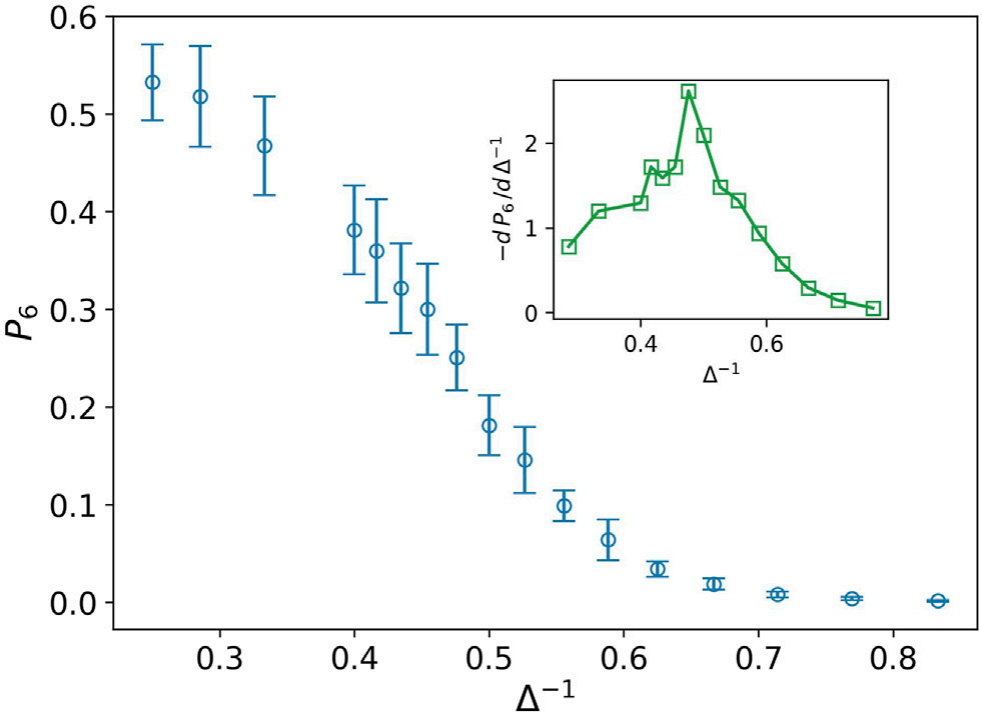
Fraction of nodes with degree six *P*_6_ (blue circle) against the inverse of attraction strength, and the attraction range has a fixed value, *σ* = 1.5. The error bars are the standard deviation of sampling over 20 simulation runs. The decreasing behavior shows lack of stiffness. The inset plot displays derivative of *P*_6_ as well. The peak splits solid-like and liquid-like phases.

Stiff nodes are those that have the most neighbors. In our case, their degree is six. *P*_6_ demonstrates the fraction of the stiff nodes in the network. We can observe in Figure 4 that *P*_6_ decreases by increasing the inverse of attraction strength in contrary to *P*_2_. Derivative of *P*_6_ has a peak at Δ^−1^ ∼ 0.476. This peak is the transition point between the two phases. On the left side, we have a network with more stiff nodes, which demonstrates a solid-like phase, and near the peak, on the right side, we expect to have a liquid-like phase.

**FIGURE 5:**
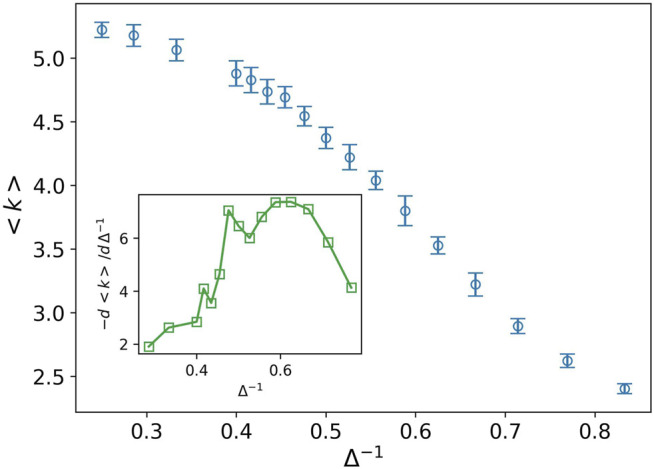
Average degree (blue circle) against the inverse of attraction strength, and the attraction range has a fixed value, *σ* = 1.5. The error bars are the standard deviation of sampling over 20 simulation runs. Two peaks are observed at almost the same points which we predict as the solid–liquid and liquid–gas transitions (see inset plot).

It is interesting that both the solid–liquid and liquid–gas transition points are observed in the plot of derivative of average degree, the inset plot in Figure 5. Indeed the average degree is a combination of all *P*_*k*_ with different weights, $<k>=\sum_{k}kP_{k}$ . In this combination, the stiff nodes are favored. Due to this fact, the transition peak for the solid–liquid is sharper than the liquid–gas transition peak.

*K*_*nn*_ displays the same behavior as the average degree, Supplementary Figure S2, a sharp peak for the solid–liquid transition and a wide peak in the liquid–gas transition point. This means that the network is homogeneous. Every node is almost connected to the neighboring nodes with the same characteristic.

If the number of components is greater than one, it means that some parts of the network are not attached to the main body of the tumor, i.e., the maximum component, and can move independently in an external chemical gradient. It may be interpreted as metastasis. The number of components per number of nodes $\rho_{c}=\frac{N_{c}}{N}$ has an increasing behavior in terms of the inverse of attraction strength, Supplementary Figure S3. Its derivative also shows the same peaks as the average degree.

Transitivity detects the ratio of the existed triangle to a possible triangle in the network and cannot exactly provide information about the low-degree nodes. Therefore, this quantity is not able to identify the liquid–gas transition as is seen in Supplementary Figure S4.

**FIGURE 6:**
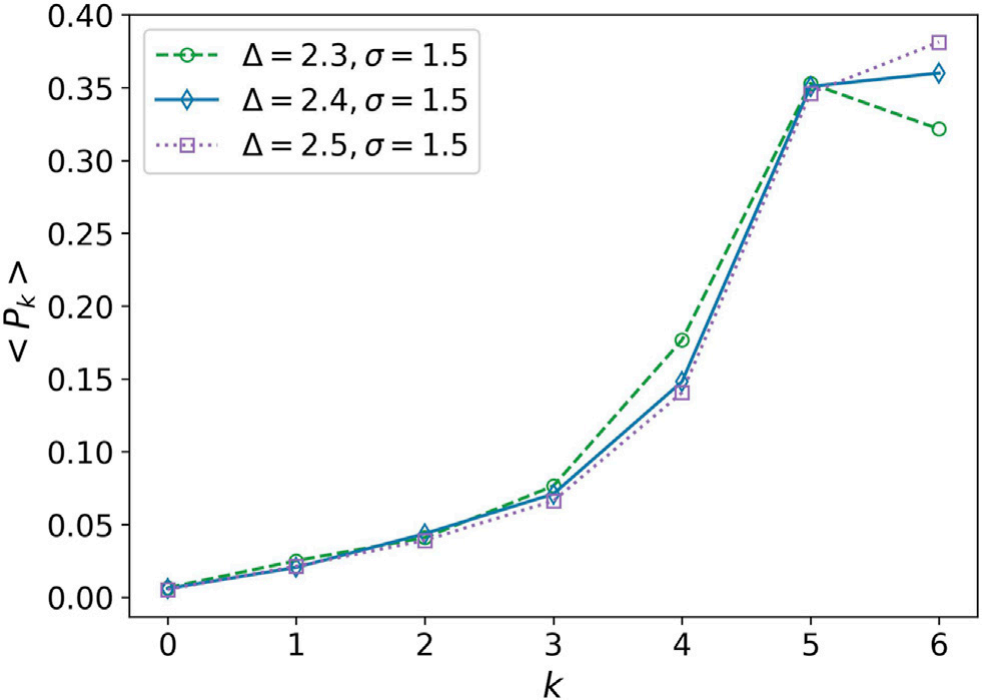
Average of the degree distribution in terms of degree. Average of the degree distribution is plotted for three values Δ = 2.3, Δ = 2.4, and Δ = 2.5. The attraction range has a fixed value, *σ* = 1.5. Every point is obtained by averaging over 20 simulation values of *P*_*k*_. The error bars are eliminated for clarity of the plots.

In derivative of the average degree of transitivity, we observe a small peak at Δ^−1^ ∼ 0.417. This peak can also be considered as a phase transition. This is the point that *k* = 6 becomes the most probable degree for the nodes, Figure 6. It is similar to change a multi-crystalline material to a single crystal, the solidity does not change, but the cells experience a kind of rearrangement. We average over the distribution function of networks obtained in 20 simulations for each parameter set for plotting this figure.

**FIGURE 7:**
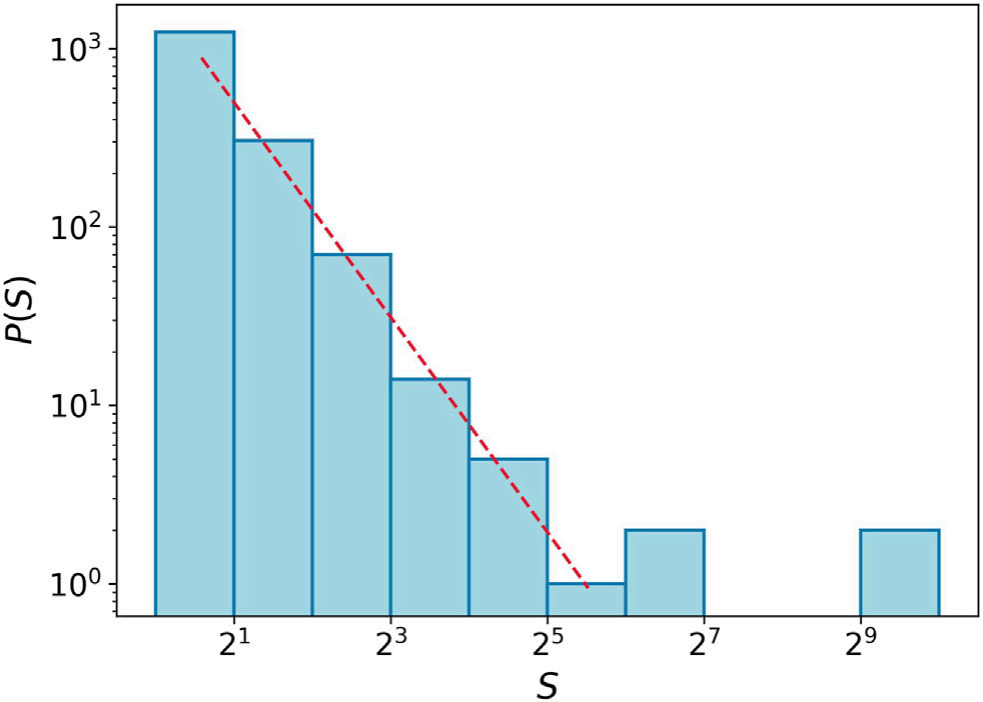
Histogram for component size at transition point Δ = 1.5. We gather data for the component size from 20 simulations. The red dashed line shows power-law function with the exponent −2.

As mentioned at the beginning of this section, we roughly talk about the solid, liquid, gas, and phase transition terms. Here, we just focus on how the tumor cells spread or how close they are to one another. Hence, it should be emphasized that there is no latent heat like quantity in such transitions, and the phases are smoothly changed to each other. In the liquid–gas transition, some criticalities are observed. For example, the histogram of component size in the critical region shows a power-law behavior. It means that the size of the components is varying by several orders of magnitude. Figure 7 depicts the histogram of the component size at Δ = 1.5 in the logarithmic scale. The red dashed line shows a power-law function with exponent −2 and is plotted for comparison. We can observe this property in an almost wide range near Δ = 1.5.

**FIGURE 8:**
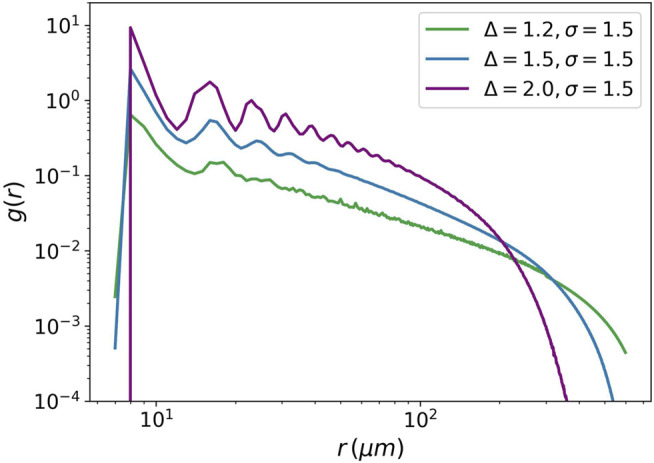
Pair distribution function against the distance between cells. The pair distribution function is plotted for three values of the attraction strength Δ = 1.2, Δ = 1.5, and Δ = 2. For all the three values, the attraction range is equal to 1.5. The power-law behavior is seen for Δ = 1.5.

The pair distribution function is another example of criticality. It measures the distribution of distances between pairs of cells in a tumor.

$$g\left(r\right)=\frac{1}{N}\underset{i,j}{\sum }<\delta \left(|r_{ij}|-r\right)>,$$ (17)

where N is the number of cells in the tumor, **r**_*ij*_ is used to show the distance between the *i*-th and *j*-th cells, by <.> we mean the ensemble averaging. We can find a power-law relationship in pair distribution function for Δ = 1.5 in the interval 60 (*μm*) to 200 (*μm*). In contrast, for two other values Δ = 2.0 and Δ = 1.2, it has exponential-like behavior or distorted power-law, respectively. Figure 8 demonstrates pair distribution function for these values.

**FIGURE 9:**
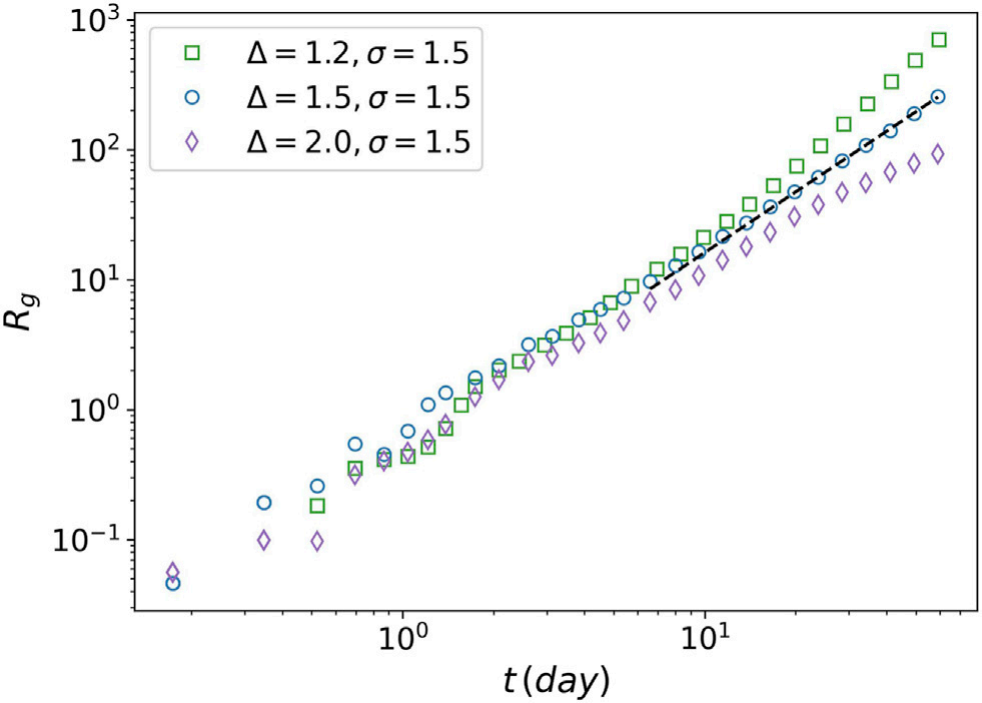
Radius of gyration of tumor against time. The radius of gyration is plotted for three values of the attraction strength Δ = 1.2, Δ = 1.5, and Δ = 2. For all the three values the attraction range is equal to 1.5. The power-law behavior is seen for Δ = 1.5 (dashed line).

Another instance of criticality is the radius of gyration, which is defined as

$$R_{g}=\sqrt{\frac{1}{N}\underset{i}{\sum }|r_{c}-r_{i}{|}^{2}},$$ (18)

where **r**_*c*_ denotes the center of mass of tumor, $r_{c}=\frac{1}{N}\sum_{i}r_{i}$ . The radius of gyration designates a characteristic length for a tumor. Figure 9 plots the radius of gyration for the three values of the attraction strength Δ = 1.2, Δ = 1.5, and Δ = 2. The power-law relationship for Δ = 1.5 is observed clearly. The tumor seems to have an unrestricted (exponential-like) growth for Δ = 1.2, while it is similar to Gompertzian growth for Δ = 2.

**FIGURE 10:**
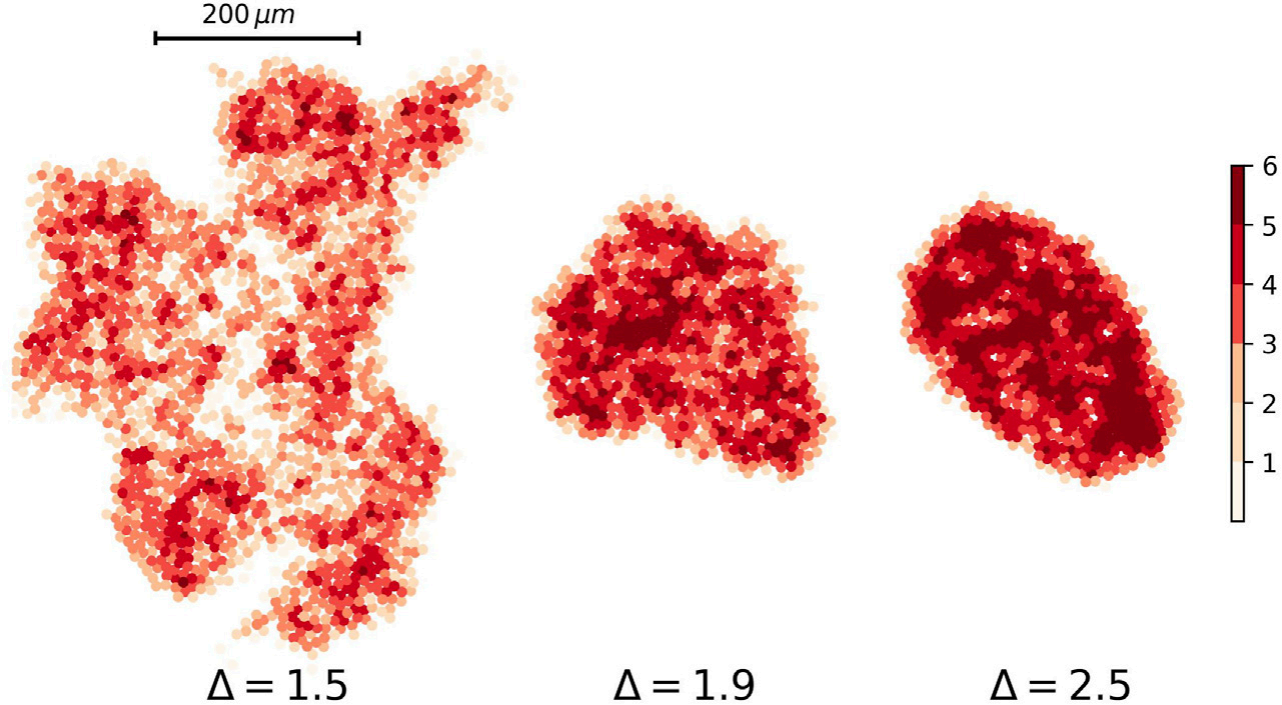
Heat plot of nodes’ degree for three different tumor networks. The attraction strength for tumors are Δ = 1.5, Δ = 1.9, and Δ = 2.5 from left to right respectively. The attraction range for all of them are the same, *σ* = 1.5.

Ultrasound, CT scan, and MRI are useful imaging methods for detecting a tumor in most tissues. They are based on the fact that points by various stiffness have a different pattern in an image. We can produce a similar image for our simulation results by assigning different color to the cells according to their neighbors in contact. Figure 10 depicts the heat plot of tumors with three values of the attraction strength Δ = 1.2, Δ = 1.5, and Δ = 2.

**FIGURE 11:**
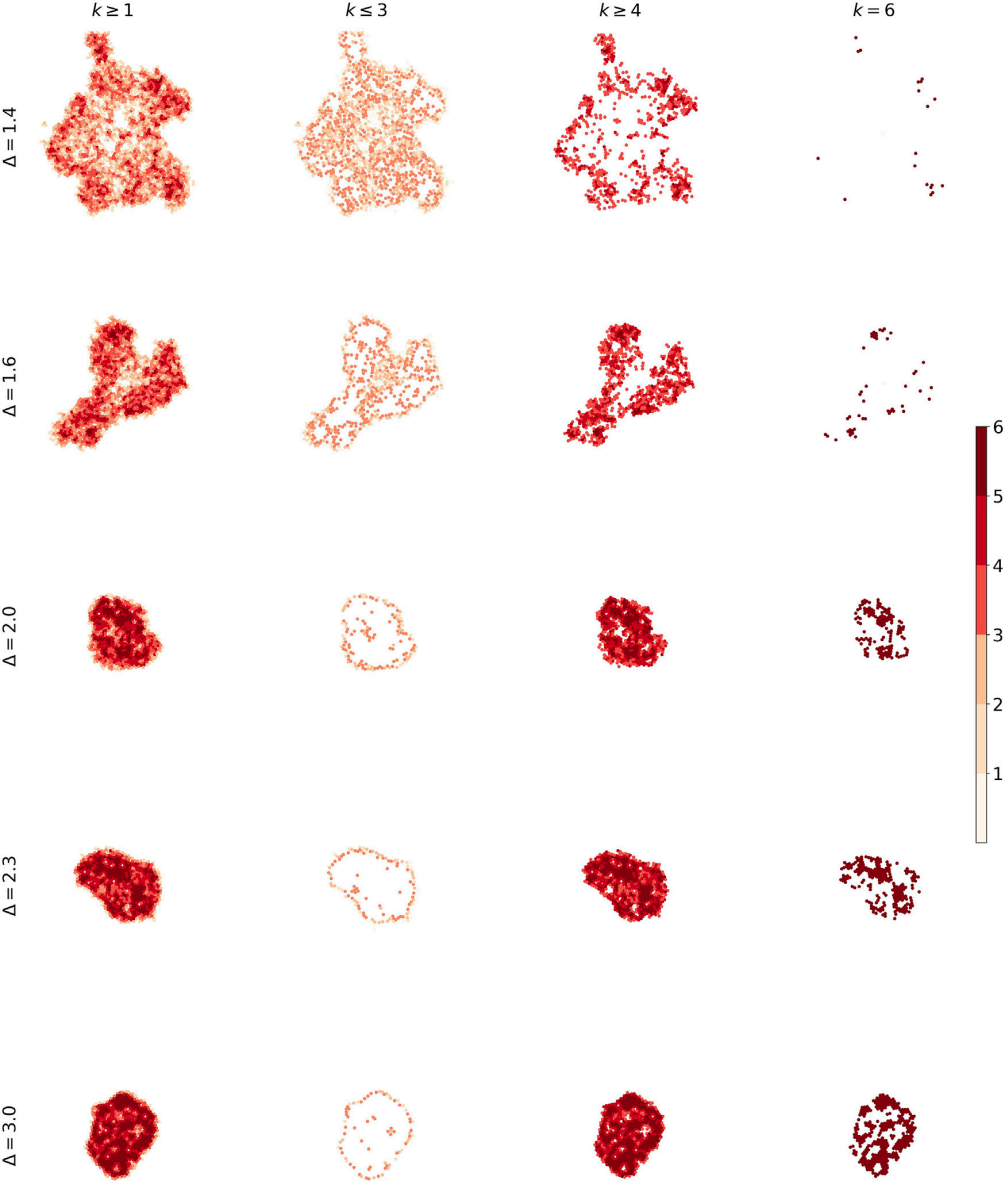
The heat plot for five tumors. We made difference between nodes according to their degrees in order to distinguish solidity of tumors.

It is useful to use a heatmap for visualizing the solidity of tumors. Figure 11 describes what we talk about different phases of the tumor. The number of low-degree nodes and high-degree nodes shows a difference between the phases.

## 4 Discussion

In this work, we present a simple scenario to explain why a tumor is benign or malignant. According to this scenario, we consider the cancerous cells as elastic disks that move randomly in the ECM, like the diffusion of particles in a viscous fluid medium. We use the Brownian dynamics to simulate such random motion. It is assumed that the shape of cells does not play an important role in the malignancy of the tumor. It should be noted that this does not contradict the EMT, which makes a cancer cell change its morphology and mobility. We can apply this alteration by changing the value of diffusivity of cells that undergo EMT. However, we do not incorporate the EMT in our model at this time, it remains for future work.

In addition to the shape of the cells, our main assumption in this work is that the cancer cells interact with each other through the secretion of chemicals. Although this is not a proven fact for cancer cells, knowing their stemness upholds it. The chemical interaction of the stem cells and progenitors in wound healing or the collective motion of nervous progenitor cells in embryogenesis is the evidence that convinces us to generalize this idea for the cancer cells. In this regard, we add a chemical short-range attractive force to the Brownian equation of motion of cells. This force tries to aggregate the cells contrary to the diffusion, which makes them spread through the ECM. Therefore, a competition exists between these two factors and in accompany with the reproduction and death of cells can explain the malignancy of a tumor. It is worth mentioning that the diffusion coefficient depends on the ECM structure and biomechanical properties. In the simulation, we fixed the diffusion coefficient value, so all the reported results will change by varying this parameter.

When diffusion becomes dominant in the system, the cells spread in the environment like the diffusion of gas molecules. In this limiting case, the number of components of the tumor is comparable with the number of cells. By increasing the attraction strength, the cells get closer together on average, and small-sized clusters are made in different parts of the system. If we do not consider the reproduction and death processes, the system undergoes an isotropic collapse, the small clusters attract each other to form the larger size clusters, and at any time, we would almost have a round tumor. But the existence of birth and death for cells modify this picture. Due to the death of a cell, a cluster may break into two or more parts. On the other hand, the birth of a new cell may lead some clusters to aggregate. In a range of attraction strengths, such as death and birth events, are important and result in having components of various sizes. Near Δ = 1.5, we have a region in which distribution of component size has power-law behavior. In this region tumor has a fractal shape, it can be confirmed by measuring the ratio of perimeter to the area of the tumor that gets very large. By passing through this region, the chemical force becomes dominants gradually, and the system has large size components that the death event could hardly break them up. The larger size components swallow the small clusters and form the overcoming clusters. The number of connection in a cluster is increased as well and the tumor becomes liquid. After that, this process continues, and the shape of the tumor is rounder than before. The degree distribution becomes more skewed to high degrees, and more than half of the nodes have degrees equal or greater than five, i.e., *k*_*med*_ = 5. This is the evidence of the solidity of the tumor. By increasing the attraction strength more and more, the system transforms to a phase with the most stiffness, and almost all the nodes connect to a maximum number of neighbors like a single crystal.

Again, we state that using the thermodynamics terminology is just for a description of the proximity of cells to each other and how they can spread. There is not any one-to-one similarity between the tumor and matter phases. For example, the liquid–gas transition of tumors seems to be a second-order phase transition, while it is a first-order for the real matter.

Finally, we should note that this is just a simple model for a description of different phases of tumors and how a metastatic event may occur. To be more realistic, the polarity of epithelial cells and EMT must be taken into account. This is one of the directions of our future work.

## Data Availability

The original contributions presented in the study are included in the article/Supplementary Materials. Further inquiries can be directed to the corresponding author.
