# Supplementary material for: Network Analysis Identifies Phase Transitions for Tumor With Interacting Cells
Source: Front Physiol. 2022 Jul 1;13:865561. doi: 10.3389/fphys.2022.865561 (PMC9283708; doi:10.3389/fphys.2022.865561)
Supplement: Supplementary file 1 [file DataSheet1.PDF]

Supplementary Material

1 SUPPLEMENTARY FIGURES

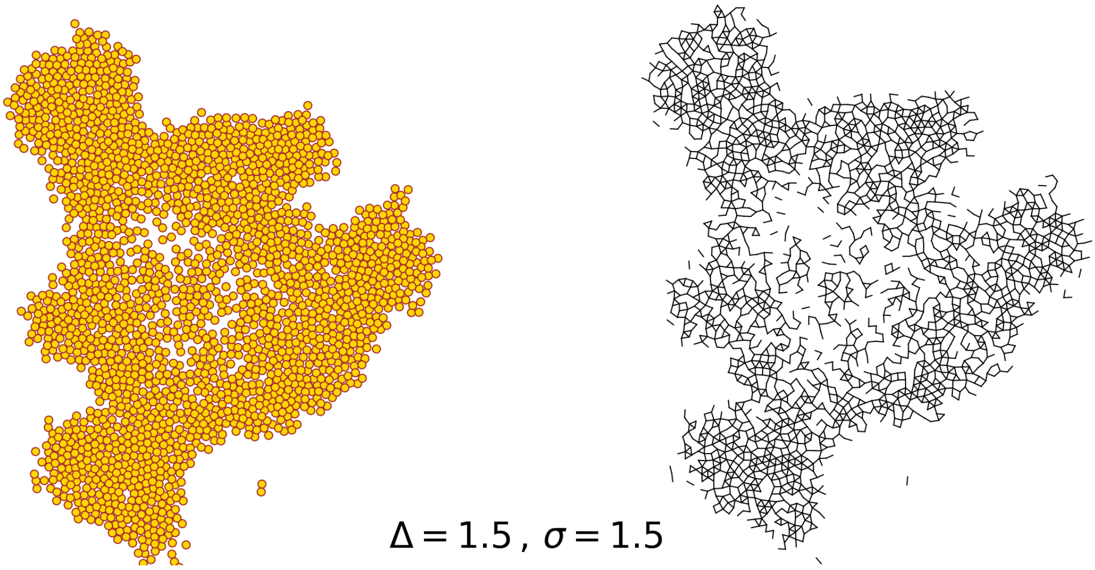

Figure S1. Image of the simulation result for tumour growth (left) and its associated network (right). The unconnected components are seen. The positions of nodes are not important, the network can be plotted in any form if its links are not cut.

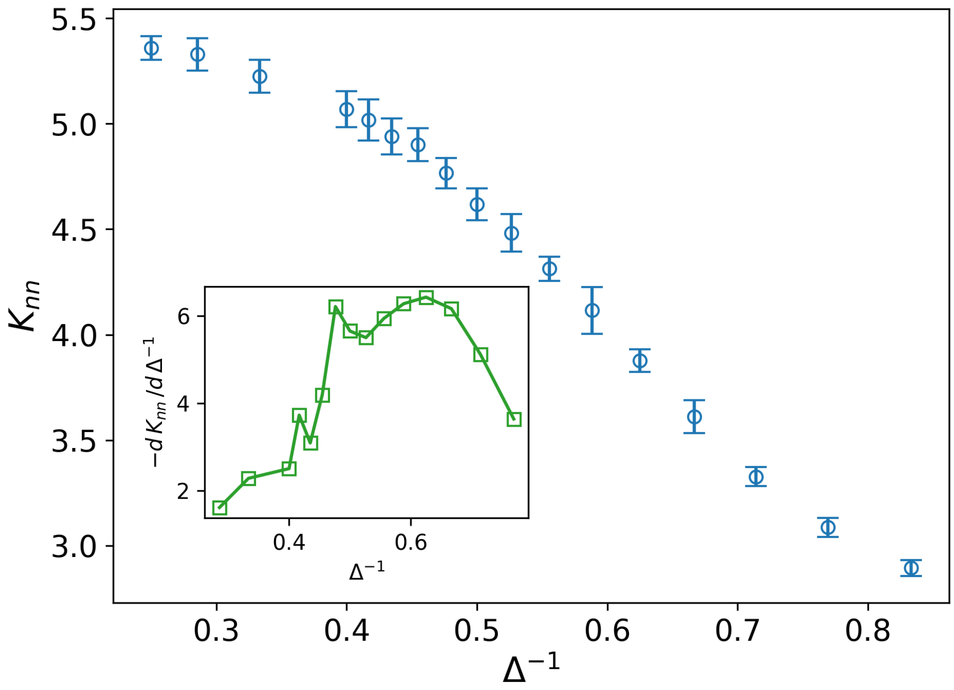

Figure S2. Average degree of neighbors (blue circle) against the inverse of attraction strength, the attraction range has a fixed value, $\sigma = 1.5$. The error bars are the standard deviation of sampling over 20 simulation runs. Two peaks are observed at almost the same points which we predict for the solid-liquid and liquid-gas transitions (see inset plot). The behavior of average degree of neighbors is very similar to the average degree.

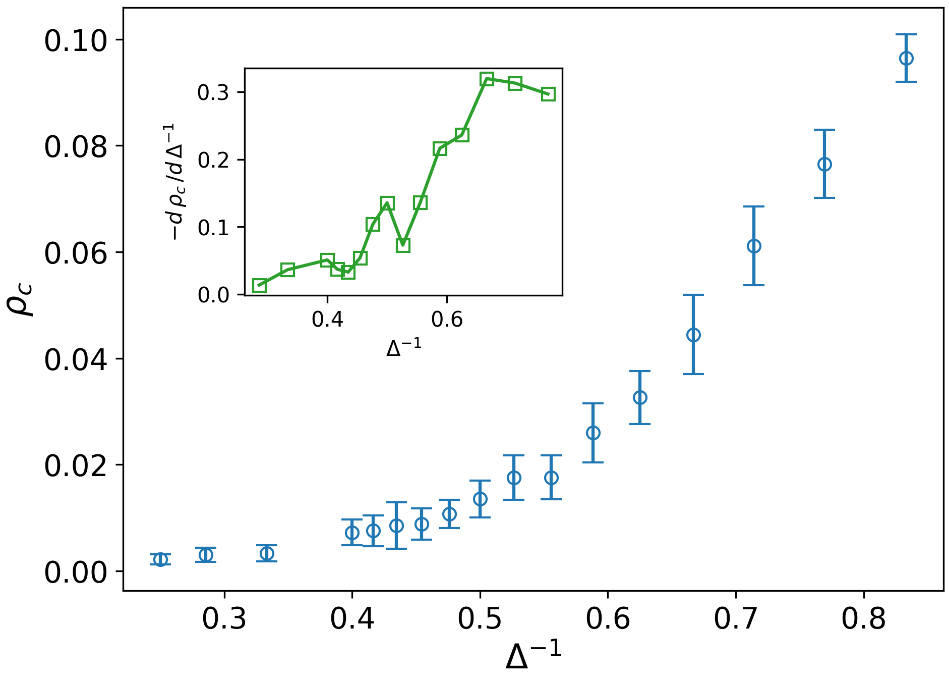

Figure S3. The number of component per number of nodes (blue circle) against the inverse of attraction strength, the attraction range has a fixed value, $\sigma = 1.5$. The error bars are the standard deviation of sampling over 20 simulation runs. Two peaks are observed at almost the same points which we predict for the solid-liquid and liquid-gas transitions (see inset plot). The behavior of average degree of neighbors is very similar to the average degree.

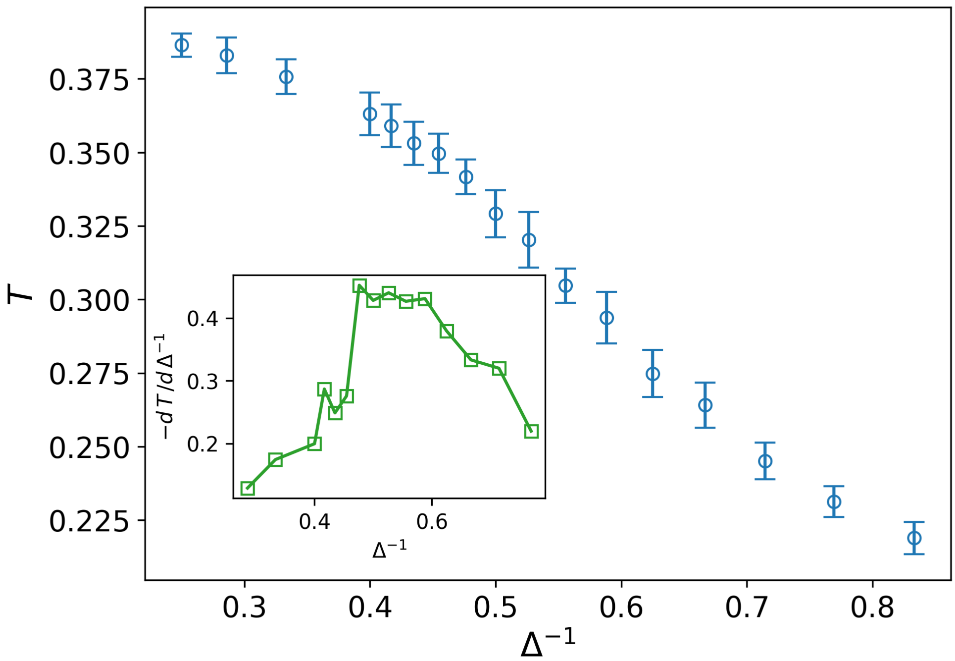

Figure S4. Transitivity (blue circle) against the inverse of attraction strength, the attraction range has a fixed value, $\sigma = 1.5$. The error bars are the standard deviation of sampling over 20 simulation runs. The liquid-gas transition peak cannot be observed due to lack of share of low degree nodes in construction of the existed triangle loops in the network (see inset plot).
